# Effectiveness of Platelet Rich Fibrin in Accelerating Canine Distal Movement: A Systematic Review of Randomized Controlled Trials

**DOI:** 10.7759/cureus.69184

**Published:** 2024-09-11

**Authors:** Swati Singh, Ravindra Kumar Jain, Abirami Selvaraj, Arthi Balasubramaniam

**Affiliations:** 1 Orthodontics, Saveetha Dental College and Hospitals, Saveetha Institute of Medical and Technical Sciences, Saveetha University, Chennai, IND; 2 Public Health Dentistry, Saveetha Dental College and Hospitals, Saveetha Institute of Medical and Technical Sciences, Saveetha University, Chennai, IND

**Keywords:** acceleration, canine retraction, dental, platelet-rich fibrin, platelet-rich plasma, tooth movement

## Abstract

This review aimed at a systematic evaluation of the available literature on the effect of platelet-rich fibrin (PRF) in accelerating canine distal movement (CD). An elaborate search of the available literature on the effect of PRF on the acceleration of canine distal movement was conducted using appropriate search terms in PubMed, Scopus, Cochrane Central, and Google Scholar until April 2024. The risk of bias within the studies was assessed using the Cochrane risk of bias (RoB)-2 tool. Publication bias was assessed with a funnel plot, and a random effects model was used for quantitative synthesis. The certainty of the available evidence was assessed with the Grading of Recommendations Assessment, Development, and Evaluation (GRADE) approach. Following the systematic search, a total of 11 studies were included for the qualitative analysis, and three studies were included for quantitative synthesis. Most of the studies reported an increased rate of canine distal movement following administration of PRF. The overall pooled CD at one, two, three, four, and five months showed a significantly increased mean CD in i-PRF (p<0.00001; MD = 0.23; 95% CI: 0.14 to 0.32), and a significantly increased rate of CD was noted during the first and second months. The available studies had a moderate to low risk of bias and a moderate certainty of evidence. Hence, with a moderate certainty of evidence and a risk of bias, it can be concluded that PRF accelerates orthodontic tooth movement during the first two months.

## Introduction and background

Orthodontic treatment with fixed mechanotherapy takes about 20 months to complete on average, and it depends on factors like the severity of the malocclusion and biomechanics, among a few of them [1]. Reduced treatment time will improve patient satisfaction and also reduce the risk of compliance issues by the patient and even treatment-related complications like root resorption, periodontal problems, and enamel demineralization [2]. Hence, procedures to reduce the treatment duration have been attempted and are in clinical practice. We can broadly classify the many methods used for accelerating tooth movement into two categories: invasive and non-invasive. Surgical methods like corticotomy, micro-osteoperforation, and piezocision require complex procedures [3]. Tooth movement has been observed to be accelerated by biological substances such as prostaglandins, vitamin D, and parathyroid hormone. Prostaglandin E1 and vitamin C administered locally produced favorable effects [4]. There have been reports of specific adverse effects following systemic dosing of these agents [5].

Platelet concentrates have high concentrations of important growth factors that promote angiogenesis, cell proliferation, and matrix remodeling; hence, they are utilized to speed up tooth movement [6]. They can be broadly classified as platelet-rich fibrin (PRF), which includes both injectable PRF (i-PRF) and leukocyte-rich PRF, and platelet-rich plasma (PRP), which can be either pure or leukocyte-rich formulations based on the cell content and fibrin architecture [7]. PRF is a second-generation platelet derivative obtained with a single centrifugation and is rich in leukocytes [8]. Injectable platelet-rich fibrin is extracted without using an anticoagulant, and when compared to PRP, it remarkably influences osteoblast behavior by affecting their proliferation, migration, and differentiation [6,9]. At low concentrations, i-PRF was the best product for bone mineralization enhancement and therefore can be preferred for bone regeneration [10].

Early on in orthodontic tooth movement (OTM), platelet concentrates (PCs) accelerate the process in the short term; their long-term effects have not yet been documented [11,12]. A previous review on PCs has reported that the available evidence is moderate to low quality with high heterogeneity [11]. Another review noted that it was challenging to draw any firm conclusions due to the wide range of studies that were available on the acceleration of tooth movement by platelet concentrates [13]. Furthermore, there are no systematic reviews that focus exclusively on how platelet-rich fibrin can hasten tooth movement. This review was undertaken to methodically evaluate the most recent research on the impact of PRF on accelerating canine tooth distal movement.

## Review

Protocol and registration

Preferred Reporting Items for Systematic Reviews and Meta-Analysis (PRISMA) guidelines were followed in conducting this review, and it was registered in the PROSPERO database using the assigned number (CRD42021261836). Additionally, the institutional scientific review board authorized the review and provided the number: SRB/SDC/ORTHO-2103/23/094. 

Population, intervention, comparison/comparator, and outcomes assessed based on eligibility requirements for study selection are listed in Table 1.

**Table 1:** Eligibility criteria for study selection PRF: platelet-rich fibrin; RCT: randomized controlled trial

Variables	Inclusion	Exclusion
Population	Clinical trials on human subjects undergoing orthodontic tooth movement	Animal studies human growing subjects. Individuals with extensive root resorption or bone resorption. Participants with any systemic illness influencing tooth mobility or those with craniofacial abnormalities. Individuals with blood disorders.
Intervention	Use of platelet rich-fibrin injectable or platelet plug to accelerate tooth movement	PRF used for any other purpose
Control	No intervention	Not applicable
Outcome assessed	Rate of canine tooth distal movement	Not applicable
Study design	RCT (split-mouth study/case-control study)	Case reports, animal studies, case series

The main result of all the included research was a comparison of the rate of tooth movement after platelet-rich fibrin (PRF) administration. 

Search strategy

An elaborate search of the available literature was conducted by PubMed, Scopus, Cochrane Central, and Google Scholar until April 2024 (Table 2). A systematic search for unpublished literature and dissertations was not performed.

**Table 2:** Search strategy for the various databases Data collection process

Database	Search terms	No. of hits
Pubmed	("platelet rich fibrin"[MeSH Terms] OR ("platelet rich"[All Fields] AND "fibrin"[All Fields]) OR "platelet rich fibrin"[All Fields] OR ("platelet"[All Fields] AND "rich"[All Fields] AND "fibrin"[All Fields]) OR "platelet rich fibrin"[All Fields] OR (("blood platelets"[MeSH Terms] OR ("blood"[All Fields] AND "platelets"[All Fields]) OR "blood platelets"[All Fields] OR "platelet"[All Fields] OR "platelets"[All Fields] OR "platelet s"[All Fields] OR "plateletes"[All Fields]) AND ("fibrin"[MeSH Terms] OR "fibrin"[All Fields] OR "fibrins"[All Fields] OR "fibrine"[All Fields]) AND ("concentrate"[All Fields] OR "concentrated"[All Fields] OR "concentrates"[All Fields] OR "concentrating"[All Fields] OR "concentration"[All Fields] OR "concentrations"[All Fields]))) AND ("tooth movement techniques"[MeSH Terms] OR ("tooth"[All Fields] AND "movement"[All Fields] AND "techniques"[All Fields]) OR "tooth movement techniques"[All Fields] OR ("tooth"[All Fields] AND "movement"[All Fields]) OR "tooth movement"[All Fields])	40
Scopus	platelet AND rich AND fibrin OR platelet AND fibrin AND orthodontic AND tooth AND movement OR canine AND retraction	110
Google Scholar	platelet-rich fibrin OR orthodontic OR tooth OR movement "canine retraction"	279
Cochrane central	‘platelet-rich fibrin’ AND ‘orthodontic tooth movement’	39

Table 2 gives the summary of the database search. The publication date, status, and language were all unrestricted. Additionally, a manual search of the qualified studies' reference lists was done.

Study selection

The titles and abstracts of the studies that were retrieved were evaluated by two writers (SS and RKJ). During the study selection process, any disagreements were discussed and addressed by discussion with the author (AB).

Data collection 

Data extraction was performed independently by two reviewers (SS and RKJ). The data collected was tabulated under the following headings: author/year/study design, study participant number, age, gender, malocclusion, intervention groups, orthodontic treatment executed, and outcome assessment methods.

Risk of bias (RoB) in individual studies

SS and RKJ used the Cochrane RoB-2 tool, which was developed by Higgins and Sterne, to independently evaluate the risk of bias present in each particular study [14,15]. Bias in individual studies is assessed in distinct domains with signaling questions focused on trial planning and execution. Risk of bias assessment of randomized controlled trials (RCTs) includes the following domains: bias resulting from the randomization process, bias due to deviations from the intended interventions (effect of assignment to intervention), bias due to missing outcome data, bias in the measurement of the outcome, and bias in the selection of the reported results.

Summary measures and synthesis of results

The subgroup meta-analysis was done by pooling studies with comparable follow-up durations. Analysis was done on the canine retraction comparison between injectable platelet-rich fibrin (i-PRF) and controls with follow-up periods of first, second, third, fourth, and fifth months. The mean differences with 95% confidence intervals (CI) were used to express the estimated effects of the intervention. The method employed was the inverse variance, and heterogeneity was assessed with I2 statistics. Values below 25% were regarded as low heterogeneity, those between 25% and 70% as moderate heterogeneity, and values beyond 70% as high heterogeneity. A significance level of p ≤ 0.05 was applied.

A funnel plot was utilized to evaluate publication bias for the studies that were included. Publication bias may be seen in the asymmetry of the studies with high standard error in the funnel plot. RevMan Web (the Cochrane Collaboration, Copenhagen, Denmark) online review authoring tool was used to analyze data from the included studies.

Additional analysis

The quality of evidence in the included studies was evaluated using the Grading of Recommendations Assessment, Development, and Evaluation (GRADE) approach. The certainty of the degree of evidence was determined by evaluating the study design, risk of bias, inconsistency, and indirectness. 

Results

Study Selection

**Figure 1:**
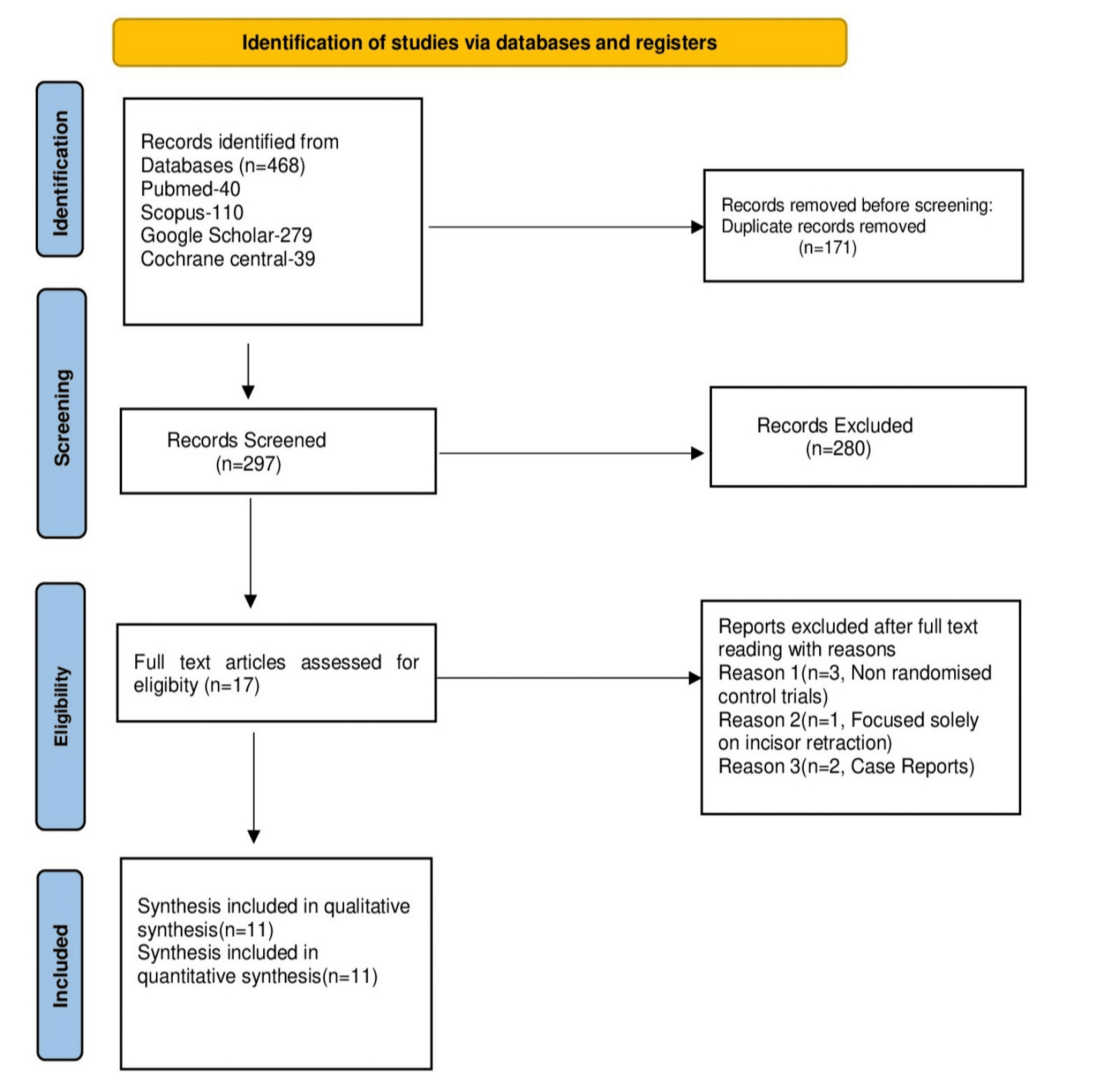
Prisma flow chart illustrating the method used to find included studies PRISMA: Preferred Reporting Items for Systematic Reviews and Meta-Analysis

On completing the search in four databases, a total of 468 studies were obtained. No studies were identified on manual search. After removing the duplicates manually, 297 studies were found eligible, which were then subjected to screening of the titles. Seventeen studies obtained were subjected to an eligibility assessment, and six of them were excluded for reasons. A total of 11 studies were included in the qualitative analysis (Figure 1).

Study Characteristics

All 11 included studies were RCTs, and a total of 300 subjects were treated with PRF. Injectable PRF was used in six of the included studies (Ammar et al., 2024; Karci et al., 2021; Erdur et al., 2021; Naji et al., 2022; Gupta et al., 2022; Zeitounlouian et al., 2021); and L-PRF plugs were used in five studies (Barhate et al., 2022; Krishna V et al., 2023; Pacheco et al., 2020; Gupta et al., 2023; Tehranchi et al., 2018) [6,16-25].

All included studies measured the rate of canine distal movement [16,18,19,23]. Digital model superimposition and manual measurements on study models were done in the included studies to measure canine tooth movement. Ten of the included trials had treatment periods ranging from eight weeks to five months except in the study by Gupta et al., 2022, in which the duration was not mentioned [22]. In all of the included papers, the calculation of samples was done. NiTi closed coil springs were used for canine retraction in most studies except in the study by Pacheco et al., 2020 E-chain was used, and in the study by Naji et al., 2022 Ricketts spring was used [19,21]. The study participants' age range was 12-28 years in most studies (Table 3).

**Table 3:** General information of the included studies Table depicting all the characteristic variables of the included studies. PRP: platelet-rich plasma; i-PRF: injectable platelet-rich fibrin; L-PRF: leukocyte-rich platelet-rich fibrin; CD: canine distal movement; OTM: orthodontic tooth movement; CI: canine inclination; RCT: randomized controlled trial

Author/year/study design	Study participant's number, age, gender	Study groups	Outcomes assessed	Orthodontic treatment involved	Study duration
Ali Mohsen Ammar, 2024 [6] RCT	60 subjects mean age: 21.1 ± 2.2 years (M:24, F: 36)	Group: PRP, Group 2: i- PRF, Group 3: Control	Rate of CD digital model superimposition	NiTi closed-coil springs applied 150 g of force on 0.019 × 0.025-inch SS archwire.	T0, T1, T2, T3, T4 (4 months)
Cagli Karci, 2021 [16] RCT	24 subjects mean age: 16.45 ± 0.27 years, 16.84 ±0.33 yrs (M: 14, F: 10)	Group 1: i-PRF, Group 2: piezocision	Rate of CD digital models measurements	Nickel-titanium closed coil springs were used on a 16x22 SS wire with 150 g of force.	T0-T6 (12 Weeks)
Uday H Barhate, 2022 [17] split-mouth RCT	15 subjects mean age: 20.73 ± 2.34 yrs (M: 0, F: 15)	Experimental group: L-PRF plugs, Control group: no intervention	Amount of CD digital model measurements	Individual canine retraction was carried out on 16x22 SS wire by NiTi closed coil spring (0.9 mm 12 mm), delivering a force of 150 g immediately after placement of L-PRF plug on the experimental side.	T0-T4 (8 weeks)
Emire Aybuke Erdur, 2021 [18] split-mouth RCT	20 subjects mean age: 21.4+/-2.9 yrs (M: 8, F: 12)	Intervention - i-PRF injection Control - sham injection	Rate of CD, digital model measurements	CD was conducted using 150 g Ni-Ti closed-coil springs on a 17x25 SS wire in both groups.	T0-T4 (12 weeks)
Rahaf Esam Naji, 2022 [19] split-mouth RCT	40 subjects allotted equally in two groups mean age: 21.3+/-1.8 yrs (M: 3, F: 37)	PRP group, iPRF group, control - saline injection	Rate of CD, study models	CD was initiated after 14 days of premolars extraction by tipping the end of Ricketts spring 3mm in the upper arch and 2mm in the lower arch to produce 150 g activation force.	21-day interval between T0 (pre-treatment) and T4
Balarama Krishna V, 2023 [20] split-mouth RCT	18 subjects (F: 18) mean age - 21.93±1.73 yrs, dropouts: 2	Intervention: L-PRF plugs, Control: no intervention	Rate of CD digital model measurements	Individual canine retraction was undertaken in both sides using sliding mechanics on 0.016″×0.022″ SS archwire with a NiTi closed coil spring (0.9 mm × 12 mm), delivering a force of 150 g.	T0 - T4 (8 weeks)
Reyes Pacheco, 2020 [21] split-mouth RCT	21 (M: 5, F: 12, dropouts: 4)	Intervention: Incorporating L-PRF membranes into the sockets, Control: absence of intervention	Rate of CD, flexible ruler	The 0.020-inch stainless steel archwire was used to distalize the canines. A 150 g force was applied using E chains.	T1 - to T5 (5 months )
Paridhi Gupta, 2022 [22] split-mouth RCT	13 subjects (M: 5, F: 8), mean age-20.6+/-3.2	Experimental: i-PRF injection, control -	Rate of CD, study model measurements	A 9 mm nickel-titanium closed coil spring with 150 g of force was placed at the control and experimental sites.	Not mentioned
Seema Gupta, 2023 [23] split-mouth, parallel-group RCT	20 subjects at T0, 16 subjects at T5 (M: 9, F: 7), mean age:21.85+/-2.45 years (4 subjects lost to follow up)	Experimental group- L -PRF plugs, Control: no intervention	Rate of CD study model photographs	To retract the canines on both sides on a 16x22 SS wire, NiTi closed-coil springs exerting at a constant force of 150 g were employed.	T0-T5 - 5 months total
Talar Zeitounlouian, 2021 [24] split mouth RCT	21 participants (6 men, 15 women) mean age-(20.85 ± 3.85 years)	Intervention group: i-PRF, Control group: no intervention	Rate of CD study model measurements	NiTi closed coil springs that were gauged to produce 150 g of force on 19 x 25 SS wire activated to begin space closure.	T0 - T5 - a total of 5 months
Tehranchi, 2018 [25] Split mouth RCT	8 subjects (M: 5, F: 3) mean age: 17.37 years, Range 12-25 years	Intervention: L-PRF, Control grp: no intervention	Rate of CD study model measurements	The canine teeth were moved distally by a NiTi closed‐coil spring on a 0.016 × 0.022‐inch SS wire.	T0 - T8 -a total of 16 weeks

RoB Within Studies

**Figure 2:**
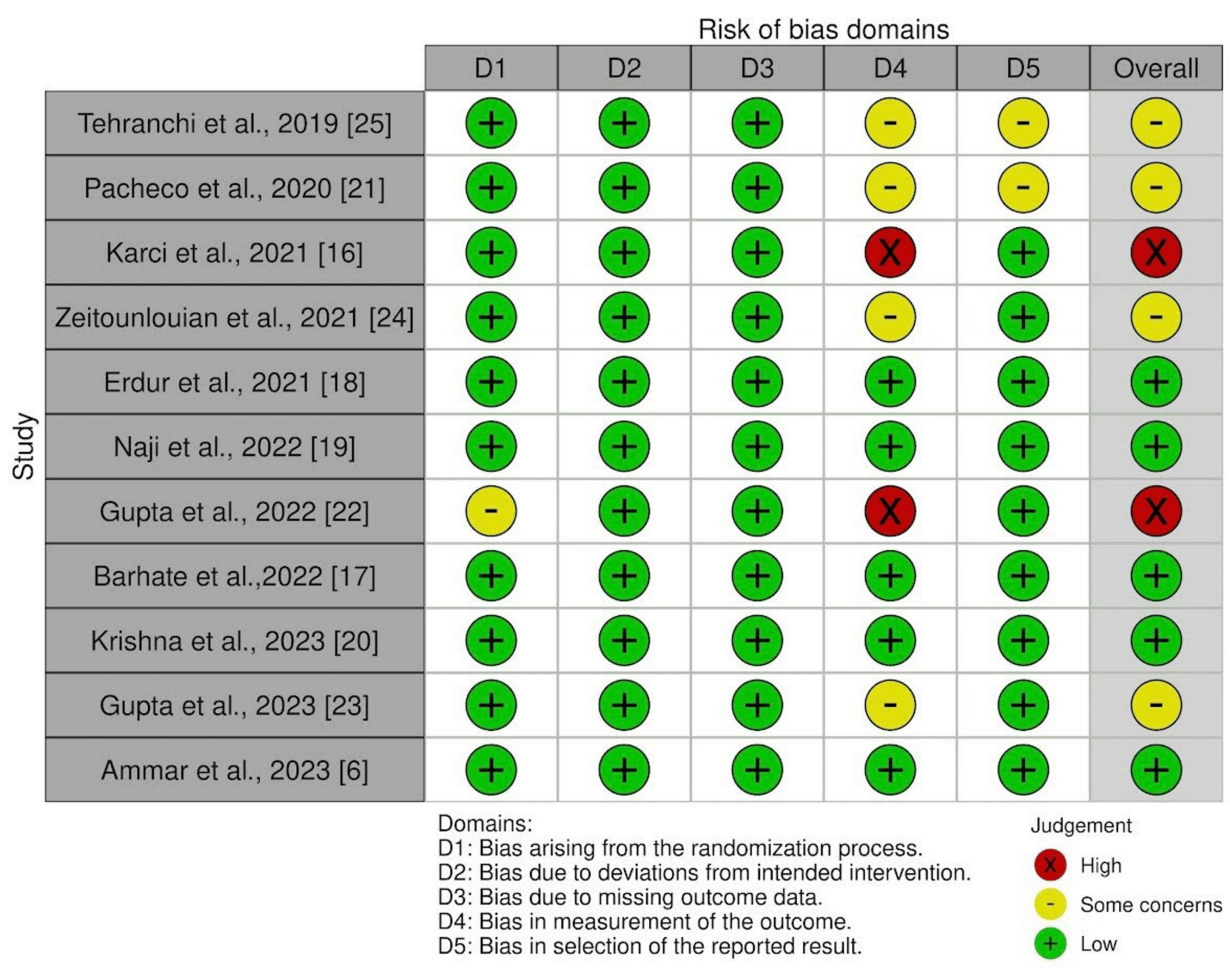
Risk of bias assessment of the included RCTs using the Cochrane risk of bias tool RCT: randomized controlled trial

**Figure 3:**
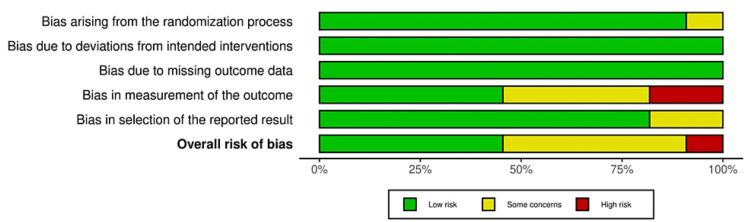
Overall risk of bias of included studies

There was low bias risk in five studies (Ammar et al., 2024; Barhate et al., 2022; Erdur et al., 2021; Naji et al., 2022; Krishna V et al., 2023) [6,17-20]. Four studies had some concerns (Pacheco et al., 2020; Gupta et al., 2023; Zeitounlouian et al., 2021; Tehranchi et al., 2018) because of bias in outcome measurements and bias in the selection of reported results [21,23-25]. Two studies had a high risk of bias (Karci et al., 2021; Gupta et al., 2022), as there was bias due to randomization and bias in outcome measurement (Figure 2, 3) [16,22].

Effect of PRF on the Rate of Canine Distal Movement

Eight out of the included 11 studies reported significant acceleration of OTM with PRF when compared to controls. Ammar et al. (2024) reported a significant acceleration of OTM with PRF over a period of four months [6]. Karci et al. (2021) noted a significant acceleration with PRF for a follow-up of three months [16]. Erdur et al. (2021) reported acceleration at all assessment time intervals over a period of 12 weeks [18]. Gupta et al. (2022) reported that the mean rate of canine retraction for i-PRF was 2.37+/- 0.56 mm/month with a statistically significant (p < 0.001) difference [22]. In the first two months, there was a noticeably higher rate of OTM. Over the course of eight weeks, Barhate et al. (2022) noted greater levels of canine retraction in the experimental sides as opposed to the control side (P = 0.001) [17]. Krishna et al. (2023) noted that the amount of canine retraction during eight weeks of follow-up was 0.28 mm more on the experimental side than the control side (P < 0.01) [20]. In both i-PRF and controls, the rate of canine distal movement reported in the included studies varied greatly.

Among the included studies, two of them reported no significant acceleration of tooth movement with PRF [19,24]. With almost similar extent of tooth movement when compared to controls. One study reported reductions in the rate of tooth movement with PRF when compared to controls [21]. 

Out of the six studies with injectable PRF as the intervention, four of them (Ammar et al., 2024; Karci et al., 2021; Erdur et al., 2021; Gupta et al., 2022) reported acceleration of CD, and out of the five studies that reported using L-PRF, four of them reported significant acceleration of CD (Barhate et al., 2022; Krishna V et al., 2023; Gupta et al., 2023; Tehranchi et al., 2018) [6,16-18,20,22,23,25]. According to Barhate et al. (2022) and Krishna et al. (2023), canines with L-PRF had a distal movement that was just 0.35 mm and 0.28 mm better than those who did not get any intervention [17,20]. After four weeks, there was no acceleration observed.

Meta-Analysis

**Figure 4:**
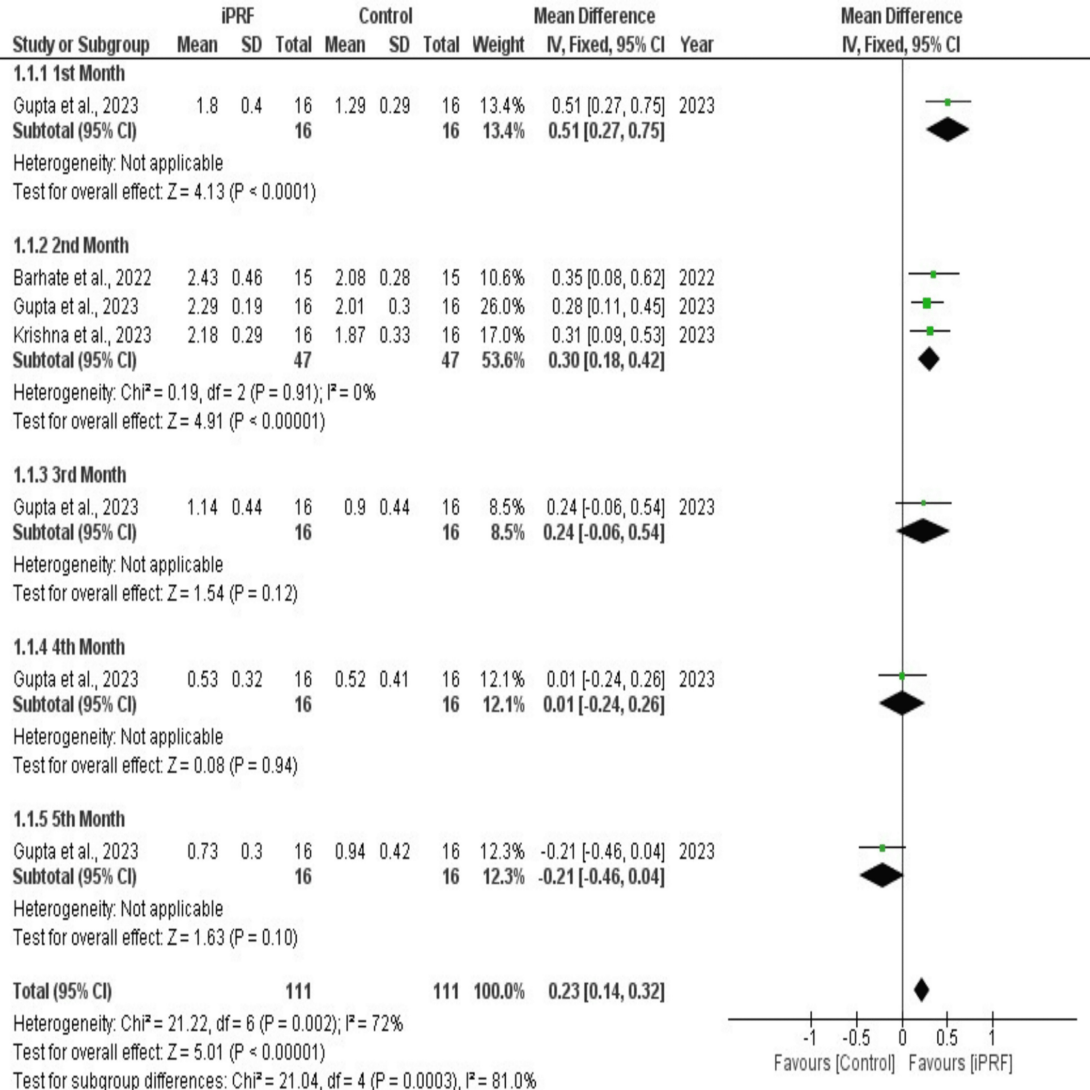
Forest plot of included studies The figure shows the forest plot for comparison of canine retraction in mm for each month. [17,20,23]

A total of three studies [17,22,23] that compared i-PRF and controls for CD at the first month, second month, third month, fourth month, and fifth month were included in the sub-group meta-analysis. There was a significantly increased CD in the first month with i-PRF (p<0.00001; MD = 0.51mm; 95% CI: 0.27 to 0.75). Similarly, i-PRF exhibited a significantly increased CD in the second month (p = 0.00001; MD = 0.30; 95% CI: 0.18 to 0.42) with no heterogeneity of I2 = 0%. In the third, fourth, and fifth months, i-PRF showed no significant increase in the rate of CD. However, the overall pooled CD at one, two, three, four, and fifth months showed a significantly increased mean CD in i-PRF (p < 0.00001; MD = 0.23; 95% CI: 0.14 to 0.32) compared to controls with a heterogeneity of I2 = 72% (Figure 4).

Publication Bias

**Figure 5:**
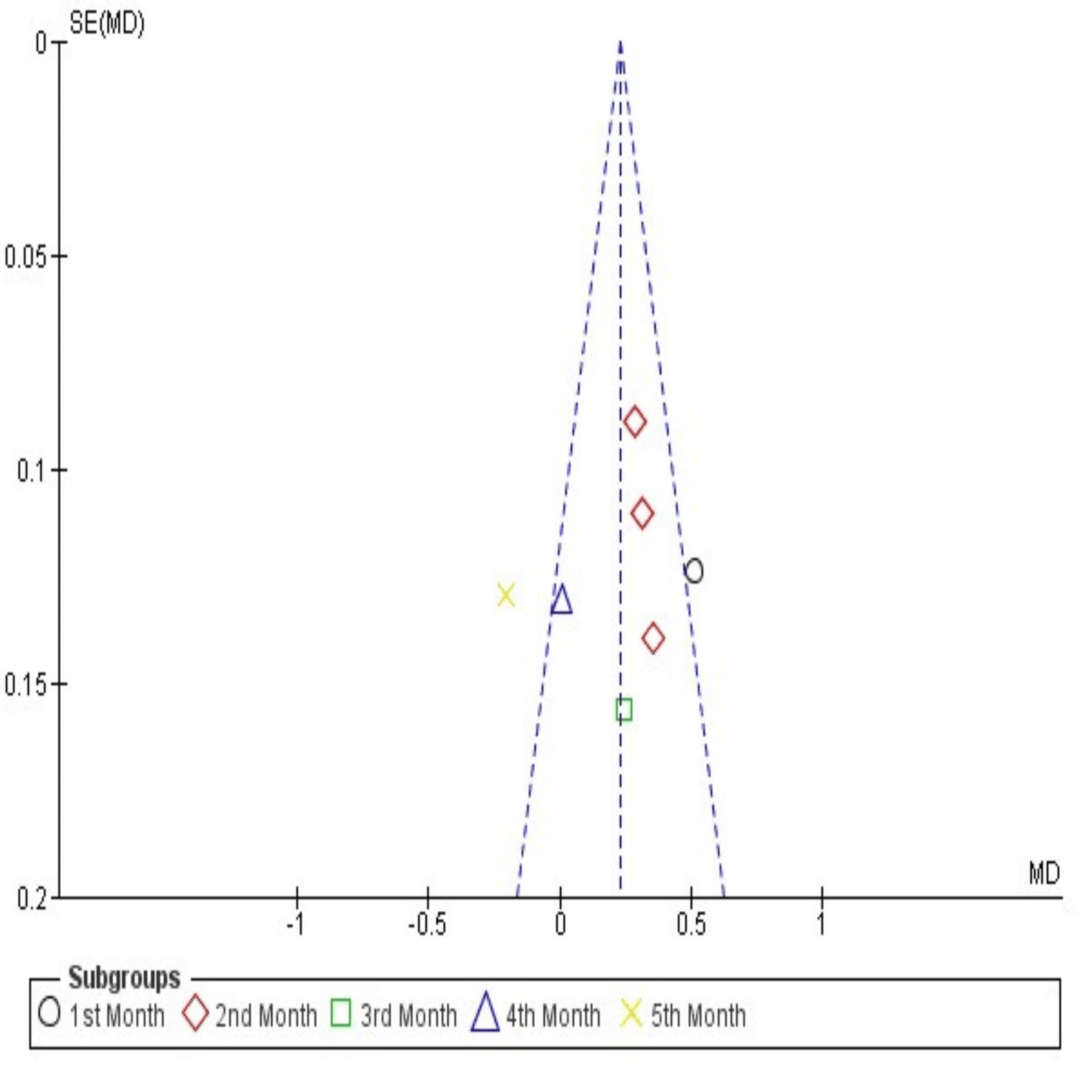
Funnel plot of included studies The amount of canine retraction in both i-PRF and control groups revealed publication bias in Gupta et al., 2023 at third month [23].

The funnel plot comparing the amount of CD in both i-PRF and control groups revealed publication bias in Gupta et al. (2023) in the third month (Figure 5) [23].

Certainty of Evidence

The overall certainty of evidence was assessed using the Grading of Recommendations Assessment, Development, and Evaluation (GRADE) approach using the GRADEpro guideline development tool (Table 4). A moderate level of certainty of the available evidence for the effect of platelet-rich fibrin on canine distal movement at various follow-up periods of the first, second, third, fourth, and fifth months was observed.

**Table 4:** GRADE pro assessment of the certainty of evidence for evidence GRADE: Grading of Recommendations Assessment, Development, and Evaluation

Certainty assessment							№ of patients		Effect		Certainty	Importance
No of studies	Study design	Risk of bias	Inconsistency	Indirectness	Imprecision	Other considerations	iPRF	Control	Relative (95% CI)	Absolute (95% CI)		
Canine retraction (Continuous)												
3	randomized trials	not serious	not serious	not serious	not serious	Strong suspect of publication bias	111	111	-	MD 0.23 higher (0.14 higher to 0.32 higher)	⨁⨁⨁◯ Moderate	IMPORTANT

Discussion

Acceleration of orthodontic tooth movement with non-surgical methods has gained popularity in recent years. One of the non-surgical methods is the use of platelet-rich concentrates (PRP, PRF) in different forms, which have been extensively researched [26]. Growth factors, enzymes, and cytokines included in platelet concentrates (PCs) may have anti-inflammatory properties that aid in tissue healing, while other cytokines, such as tissue necrosis factor (TNFs), may increase inflammation and hasten the OTM [27]. The balance between osteoclasts and osteoblasts may be influenced by PCs decreasing turnover and inducing bone formation. It is proven that there is a slow release of growth factors and enzymes in PRF as compared to PRP. The concentration and content of growth factors and their timing of release will determine the effect of PRF, which is usually both pro-inflammatory and anti-inflammatory [28]. It has also been reported that L-PRF has long-term release of growth factors with higher amounts of released TGF-β1, leading to a stronger induction of cell migration. Animal experiments have confirmed a more pronounced acceleratory effect of i-PRF on OTM than PRP.

Previous published systematic reviews (SRs) have reported on the effects of all platelet concentrates combined, but there are not many SRs specifically on PRF. Hence the present study was aimed at reviewing the available literature on the effect of PRF for acceleration of orthodontic tooth movement.

Summary of evidence

Most of the studies included in this review reported an acceleration of canine distal tooth movement after using PRF (L-PRF, i-PRF) for the evaluated period. On quantitative analysis, the effect of PRF on the acceleration of canine distal movement was significantly higher during the first and second months, and also an overall acceleration of canine distal movement was observed. The included studies had a moderate to low risk of bias, and all were randomized controlled trials. A moderate certainty of evidence was observed with the GRADE approach confirming the acceleration of canine distal movement with PRF.

The monthly increase in canine distal movement with both PRF and controls was documented in the studies that were part of the meta-analysis. The data of CD in mm was pooled for the meta-analysis. There were many methodological differences in the pooled studies, as some had used i-PRF and some had used L-PRF plugs. The methods of preparing PRF were different, and the dosage and frequency of i-PRF administration were also different in the included studies. Zeitounlouian et al. (2021) repeated the injection after one month; Erdur et al. (2021) injected immediately after the extraction, and after 15 days; Ammar et al. (2024) injected PRF immediately before retraction, and eight weeks later it was repeated [24,18,6]. On the other hand, Barhate et al., 2022; Krishna V et al., 2023; and Gupta et al., 2023, all used an L-PRF plug, which was prepared by the same method and was placed immediately after extraction of the premolars and was sutured [17,20,23]. Hence, for the meta-analysis, a random effects model was chosen for the evaluation of CD in the first, second, third, fourth, and fifth months. A meta-analysis for comparison of i-PRF and L-PRF was not performed in this review owing to the differences in study methods and duration.

PRF, unlike PRP, has a sustained effect on tooth movement, as most studies have reported significant acceleration until the time of evaluation. PRF has a fibrin network and a three-dimensional organization and can store the proteins within it, allowing the slower release of GFs over time. There is also a greater incorporation of circulating cytokines into the fibrin meshes, which explains the long-term effect. iPRF significantly increases the expression of inflammatory mediators like IL-1b, MMP8, and RANKL, while the orthopantomagram (OPG) values decrease significantly. As a result, osteoclast migration and activity are promoted.

PCs in general promote the acceleration of OTM, but various published systematic reviews have given different conclusions. The study reported (SR) by Katyal et al. has concluded no significant acceleration of OTM by PCs; the SR by Yao et al. has concluded a significant short-term acceleration of tooth movement; Lorente et al. pointed out that there was a tendency for accelerated tooth movement with PC when used immediately after premolar extraction and retraction force given immediately [11,12,13]. 

A low confidence level of evidence exists to support the acceleration impact of PCs, as reported in a recent systematic review [29]. In the current review, we noticed acceleration with PRF (both i-PRF and L-PRF) at the first and second months, and they reported acceleration of OTM with i-PRF only during the second month as well as an overall acceleration. The meta-analysis of the current SR contained more recent research; six studies in total were included for the meta-analysis of the second month, and four studies were included for the meta-analyses of the first and third months.

The effect of PRF on tooth movement has been studied in the systematic study by Sharan et al., which is similar to the current review. [30] According to their study, there was moderate evidence to suggest that PRF accelerated OTM, which is similar to the present study, but in the present review with the quantitative synthesis, we observed a significant effect on the acceleration of OTM with PRF during the first two months with a moderate risk of bias and a moderate certainty. The meta-analysis and the GRADE analysis led to different review opinions. Although the other studies conducted a meta-analysis of the derived rate of canine retraction, which was gained from the data mentioned in those studies, we conducted the meta-analysis utilizing the data of canine retraction at monthly/weekly intervals as described in the included studies.

Limitations 

Outcome measures were not uniform in the included studies, which is a major limitation. Although some studies had a moderate RoB limiting the quality of evidence, two studies had a high risk of bias. A systematic search for unpublished literature and theses was not performed. The samples in some of the included were very few, and this in turn affects the validity of the results. Hence, good quality studies with a larger sample size and a robust methodology are required so that we can come to a consensus on the dosage and form of PRF that can be recommended for clinical practice.

## Conclusions

Considering the limitations of this review, it can be concluded that the use of platelet-rich fibrin was effective for the acceleration of canine teeth distal movement. A moderate certainty of evidence is available with a moderate risk of bias, which supports the role of platelet-rich fibrin in accelerating canine distal movement during the first two months. Even though individual study differences were evident and some studies had a high risk of bias, there was an overall acceleration of canine distal movement observed while using platelet-rich fibrin either as injectable or as fibrin plugs. Hence, platelet-rich fibrin can be used in routine clinical orthodontic practice whenever an acceleration of canine tooth distal movement is desired.
